# Predictive analysis of transmissible quinolone resistance indicates *Stenotrophomonas maltophilia *as a potential source of a novel family of Qnr determinants

**DOI:** 10.1186/1471-2180-8-148

**Published:** 2008-09-16

**Authors:** María B Sánchez, Alvaro Hernández, José M Rodríguez-Martínez, Luis Martínez-Martínez, José L Martínez

**Affiliations:** 1 Departamento de Biotecnología Microbiana, Centro Nacional de Biotecnología, CSIC, Darwin 3, Cantoblanco, 28049-Madrid, and CIBERESP. Spain; 2 Departamento de Microbiología, Universidad de Sevilla, Av. Sanchez Pizjuan SN 41009-Sevilla, Spain; 3 Service of Microbiology, University Hospital Marqués de Valdecilla, Santander, Spain; 4 Department of Molecular Biology, University of Cantabria, Santander, Spain

## Abstract

**Background:**

Predicting antibiotic resistance before it emerges at clinical settings constitutes a novel approach for preventing and fighting resistance of bacterial pathogens. To analyse the possibility that novel plasmid-encoded quinolone resistance determinants (Qnr) can emerge and disseminate among bacterial pathogens, we searched the presence of those elements in nearly 1000 bacterial genomes and metagenomes.

**Results:**

We have found a number of novel potential *qnr *genes in the chromosomes of aquatic bacteria and in metagenomes from marine organisms. Functional studies of the *Stenotrophomonas maltophilia *Sm*qnr *gene show that plasmid-encoded SmQnr confers quinolone resistance upon its expression in a heterologous host.

**Conclusion:**

Altogether, the data presented in our work support the notion that predictive studies on antibiotic resistance are feasible, using currently available information on bacterial genomes and with the aid of bioinformatic and functional tools. Our results confirm that aquatic bacteria can be the origin of plasmid-encoded Qnr, and highlight the potential role of *S. maltophilia *as a source of novel Qnr determinants.

## Background

Quinolones are synthetic antibiotics; therefore, it was thought that the existence of transferable quinolone resistance genes in nature would be unlikely. Resistance mechanisms for these drugs were expected to be only chromosomally encoded. It was believed that only mutations in the quinolone targets (DNA gyrase or topoisomerase IV) or mutations that led to a permeability decrease and/or overproduction of multidrug resistance (MDR) efflux pumps would result in resistance to these antibiotics [1,2].

It was thus assumed that resistance to quinolones could not spread as the consequence of horizontal gene transfer (HGT). Later on, the acquisition of quinolone resistance due to HGT was proposed as a possibility based on *in vitro *experiments [3]. This was later confirmed in 1998, with the description of a plasmid-encoded quinolone resistance determinant, that was named Qnr, in clinical isolates [4]. More recently two other transferable quinolone resistance determinants have been described, the bifunctional aminoglycoside/quinolones inactivating aminoglycoside acetyltransferase AAC(6')-Ib-cr [5,6] and the quinolone efflux determinant, QepA [7,8].

Since the discovery of Qnr, the presence of different *qnr *families (A, B, S) in resistance plasmids [9] has been found worldwide in different bacterial pathogens [10-15]. More recently, plasmid-encoded Qnr determinants have also been described in environmental isolates of *Aeromonas *spp. [16]. The presence of Qnr genes in chromosomes has also been shown, including QnrA in *Shewanella algae*, which is likely the origin of plasmid-encoded QnrA determinants [17], and different members of the Qnr family from *Vibrionaceae *species [18,19].

The Qnr proteins belong to the pentapeptide repeat protein (PRP) family, which is defined by the presence of repetitions in tandem of the pattern (A/C/S/T/V)(D/N)(L/F)(S/T/R)(G/R) [20-22,17]. A characteristic feature of the Qnr proteins is that they are formed by two domains of pentapeptide repeats separated by a single glycine. This structure matches a motif of unknown function named Cluster of Orthologous Group of Proteins (COG) 1357 . Even though members of the PRP family have been identified both in prokaryotes and eukaryotes [22], *qnr *genes presenting the above described COG1357 motif have a much narrower distribution, mainly in plasmids and in the chromosomes of some bacterial species. The amino acid identity among different Qnr proteins families rates between 39–60% [23].

Some studies have demonstrated that Qnr protects both gyrase and topoisomerase IV from the activity of quinolones [15,24-26]. Other members of the PRP family, that may play a similar role include McbG, which protects microcin B17-producing bacteria from the activity of this DNA replication inhibitor [27] and MfpA [28], a protein that most likely binds GyrA [22] and provides low-level quinolone resistance to *Mycobacterium tuberculosis*.

Although *qnr*-like elements that contribute to intrinsic quinolone resistance have been described in the chromosome of *Enterococcus faecalis *[24] and more recently in other Gram-positive bacteria [29], their homology with *qnr *genes from Gram-negative bacteria is low (around 25%), so that this family of resistance elements has not been included in our analysis.

It is important to note that plasmid-encoded quinolone resistance is more prevalent than expected considering the date of the first isolation, which suggests that these plasmids have been circulating for some time before they were first described [30]. This highlights the need to implement methods to predict resistance before it is recognized within clinical settings.

A methodology for predicting the possibility of emergence of a new mechanism of resistance before it appears in bacterial pathogens has been recently proposed [31]. The availability of sequenced genomes allows this type of analysis. This information was used to search for the presence of Qnr determinants in available sequenced bacterial genomes and metagenomes. A putative *qnr *gene present in the chromosome of the opportunistic pathogen *Stenotrophomonas maltophilia *was chosen to perform functional analyses. *S. maltophilia *is a nosocomial pathogen intrinsically resistant to several antimicrobials [32] due to the activity of antibiotic-inactivating enzymes [33-36] and MDR efflux pumps [37-43].

The possibility that chromosomally-encoded *S. maltophilia qnr *gene (hereafter named as Sm*qnr*) could be functional in a heterologous host has been explored. As the result of our work, we have described new *qnr *genes in the chromosomes of sequenced bacterial genomes and have found that the Sm*qnr *gene from *S. maltophilia *renders a low-level quinolone resistance phenotype upon its expression in *Escherichia coli*. This study demonstrates the feasibility of utilizing currently available databases along with bioinformatics and functional tools as an approach for predicting resistance before it emerges in human pathogens.

## Methods

### Bioinformatic tools and nomenclature of predicted Qnr proteins

Multiple protein sequence alignments were carried out with the program ClustalW2 [56]. Similarity search was performed with the program BLAST (NCBI) [57] and genomicBlast . Identification of conserved motifs [58] ([59] was done by using Conserved Domain Database and Search Service at . The Qnr proteins alignments and homology trees were obtained using CLUSTALW2 and Jalview alignment editor with default parameters [60]. Homology tree was calculated using the method "Average distance using percentage of identity" .

The predicted Qnr proteins were named following the rules proposed in [61].

### Bacterial strains and growth conditions

The bacterial strains and plasmids used are shown in Table 1. The strains were grown in Luria-Bertani (LB) broth [62] at 37°C unless otherwise specified.

### Susceptibility antibiotic assays

The susceptibility assays for the different antibiotics were performed in Mueller Hinton broth (Pronadisa) plus Isopropyl-thio-ß-D-galactopyranoside 0.5 mM, using the twofold dilution method in 96-well microtiter plates. The results were recorded after 48 h of incubation at 37°C. To ensure that the observed changes were consistent, all Minimal Inhibitory Concentrations (MICs) were determined in three independent assays, using different bacterial cultures on different days. In all cases, a control strain containing the plasmid pGEM-T without any insert was included in MICs determinations. In most cases, there were not inter-assay differences in the MIC values. In a few cases, there were one-dilution differences. For the latter, the assay was repeated one more time to further assure assay reliability.

The quinolones used were ciprofloxacin, enoxacin, garenoxacin, grepafloxacin, levofloxacin, moxifloxacin, nalidixic acid, norfloxacin, trovafloxacin and sparfloxacin.

### DNA manipulations

The genomic DNA was extracted using the GNOME^® ^DNA Kit (Q-BIOgene). The PCR Master Mix (Promega) was used to amplify full-length Sm*qnr *genes from the different *S. maltophilia *strains without their promoter sequences. The reaction contained 100 ng of genomic DNA of each *S. maltophilia *isolate as template, and 1 μM of two different sets of specific Sm*qnr*-primers, QnrM+ (5'-CTTGGCATGGAATCCCTGAT-3')/QnrM-(5'-TGATGCCTACGGCACCAC-3') and QnrMR55+ (5'-CATGGCATGGAATCCCCGAT-3')/QnrMR55-(5'-TGATGTCTACGGCACCAC-3'). We used two sets of primers because the regions around *qnr *are slightly different in the sequenced *S. maltophilia *strains K279a and R551-3. The reactions had one denaturation step at 94°C for 5 minutes, followed by 35 amplification cycles: 94°C for 30 seconds, 61°C for 45 seconds for annealing, and 72°C for 1 minute for elongation, with a final extension step of 72°C for 5 minutes. The PCR products (660 bp) obtained from the different *S. maltophilia *strains (Table 1) were electrophoresed in 1% agarose gels with TAE, purified from the gel with GFX™ PCR DNA and Gel Band Purification Kit (GE Healthcare) and cloned in pGEM-T plasmid (Promega) generating the corresponding recombinant plasmids (Table 1). The transformation of *E. coli *KZM120 was made as described [63]. We chose this particular *E. coli *strain because it lacks the major *E. coli *MDR pump and it has been shown previously that the utilization of MDR-defective strains facilitates the characterization of low-level mechanisms of resistance [39]. The transformants were selected in LB agar with 100 μg/ml carbenicillin. The recombinant plasmids were purified using Wizard^® ^Plus SV Miniprep Kit (Promega) and the insert of each plasmid was sequenced using the universal primers M13 forward and M13 reverse by Secugen S.L.  to confirm the identity of the sequence and establish the orientation of the *qnr *gene. The sequences of the different Sm*qnr *genes have been deposited at GenBank with numbers from EU681371 to EU681385. Only those plasmids containing the Sm*qnr *gene in the right orientation to allow expression from the pGEM-T *lac *promoter were used in the functional assays.

### Identification of Smqnr in different S. maltophilia isolates

The potential presence of Sm*qnr *in both clinical and environmental *S. maltophilia *strains was evaluated by PCR using the primers qnrI1 (5'-AGAAAGTGGTCGACCAGCAG-3')/qnrI2 (5'-GCAGGTTCGACTTCTTGATG-3') and qnrI3 (5'-CAACGCCAGCTTCATGAACC-3')/qnrI4 (5'-AGTTGGCGCTGTTCCAGTCG-3'), which amplify 312 bp and 220 bp fragments of two internal regions in the Sm*qnr *gene of *S. maltophilia *respectively.

The PCR was made as described above, with the following program, one denaturation cycle at 94°C for 5 minutes, followed by 35 amplification cycles: 94°C for 30 seconds, 55°C for 45 seconds for annealing, and 72°C for 30 seconds for polymerization, with a final extension of 72°C for 5 minutes. The PCR products were analyzed in 1% agarose gels with TAE.

### Expression and identification of the SmQnr protein

The amount of SmQnr protein was estimated by SDS-PAGE [63] and Coomassie blue staining, using cell extracts from bacteria grown in LB at 37°C overnight. The amount of proteins loaded in each lane was normalized to around 4 × 10^7 ^cells. In all cases, the global amount of proteins was equal in all lanes of a given gel as can be seen after staining. This serves as a supplementary loading control [64].

To ensure that the protein with the predicted molecular size was indeed SmQnr, the corresponding band was excised and identified, as described [65,39] by Peptide Mass Fingerprinting using MS-MALDI TOF (Proteored: ).

### Ethical considerations

This investigation did not require ethical clearance.

## Results and Discussion

The identification in environmental microorganisms of putative antibiotic resistance elements [44] that might transfer in the near future to pathogenic bacteria is an important topic that we are just beginning to address [31]. To that goal a combination of bioinformatic and functional tools can give information to predict resistance before it emerges. Following this concept, we have explored the presence of putative *qnr *genes in available databases of bacterial genome sequences, because the genetic context of plasmidic *qnr *genes supports the idea that they have been acquired by HGT. For instance, the plasmid-encoded *qnrA *gene has been found in a *sul1*-type integron near an ISCR1 element [21,45,25]. A similar structure was found in *qnrB2 *[46], and *qnrS *is adjacent to a Tn3 transposon structure [10,11]. In contrast, it has been reported that chromosomal *qnrA*, in *Shewanella algae *[17] and several *qnr*-like genes identified within the *Vibrionacea *family [18] are not linked to these mobile genetic elements.

This suggests that chromosomally-encoded *qnr *genes have not been acquired recently by HGT because of recent antibiotic selective pressure and that yet unidentified chromosomally-encoded *qnr *genes could be a source of new transferable quinolone resistance genes.

### Bioinformatic search of putative qnr genes

**Figure 1:**
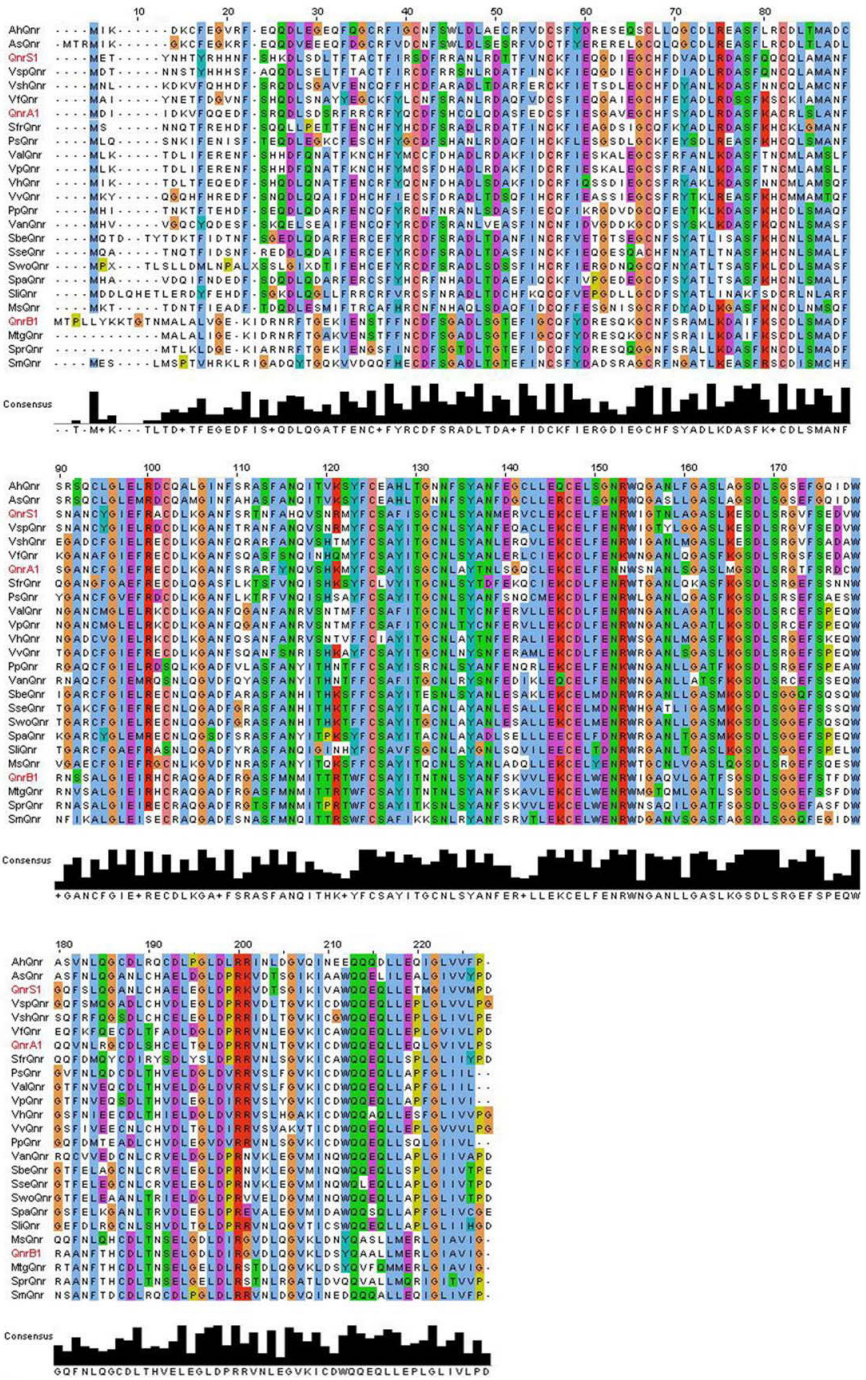
**Homology of predicted new chromosomally-encoded Qnr proteins**. The Figure shows the alignment of chromosomally-encoded putative Qnr proteins from the analysis of around 1000 genomes and metagenomes. The figure includes one member of each plasmidic Qnr protein family (highlighted in red), so that the similarities are clearer. The putative Qnr protein from the sea metagenome is also included (MtgQnr). As shown in the Figure, conservation is high along the proteins, although the similarities are lower at their N-terminus. The automated annotation of Swo*qnr *presents two stop codons. Since we ignore whether this is a sequence mistake or Swo*qnr *is a pseudogene, a manual translation of its nucleotide sequence was performed for this alignment. Stop codons are represented with an X. Accession numbers of the proteins used for the alignment are: AhQnr (YP_854820.1), AsQnr (YP_001143795.1), MsQnr (ZP_01897167.1), QnrA1 (ACA43024), QnrB1 (ABG82188), QnrS1 (ABU86826), SbeQnr (ZP_02157732.1), SfrQnr (YP_750786.1), SliQnr (ABO22341.1), SmQnr (ZP_01643096.1), SpaQnr (ABV89003.1), SprQnr (YP_001478290.1), SseQnr (ABV35251.1), SwoQnr (ZP_01539189.1), PpQnr (CAG21998.1), PsQnr (EAS39797.1), ValQnr (ZP_01261394.1), VanQnr (ZP_01234687.1), VfQnr (ZP_02136549.1), VhQnr (ZP_01985396.1), VpQnr (ZP_01990287.1), VshQnr (ZP_01866232.1), VspQnr (ZP_00989608.1), VvQnr (AAO07889.1) and MtgQnr (AACY020347520).

To address the presence of putative *qnr *genes in the chromosomes of bacteria that could serve as reservoirs of this family of quinolone resistance determinants, a bioinformatic iterative search by sequence homology to several alleles of QnrA, QnrB and QnrS was conducted against genomic or Whole Genome Shotgun Sequence (WGS) databases at the NCBI home page . Most of the putative *qnr *genes found in this search have been previously annotated as hypothetical proteins containing the COG1357 motif. As shown in Figure 1, chromosomally-encoded Qnr proteins present a large degree of homology.

**Figure 2:**
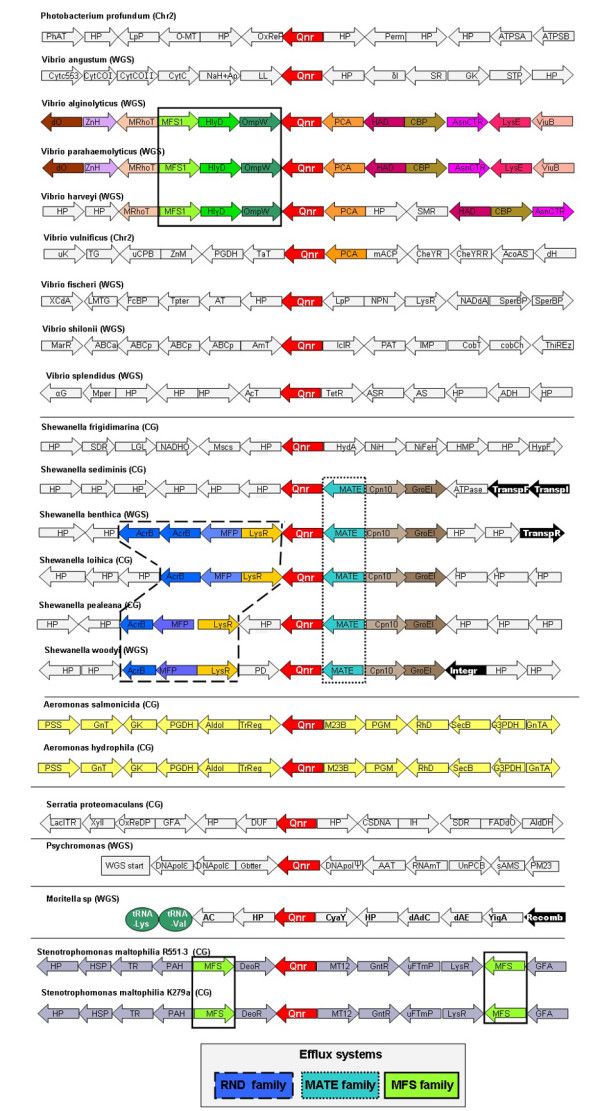
**Predicted *qnr *genes in genome databases**. The structure of the regions surrounding predicted *qnr *genes (in red) is shown. Elements that might contribute to transfer of those genes are highlighted in black. The presence of putative MDR systems is also highlighted. WGS (whole genome shotgun), CG (Complete genome), Chr (Chromosome). Only those putative *qnr *determinants showing more than 45% identity with known plasmid-encoded QnrA or QnrB determinants were included in the analysis. The abbreviations used in the Figure are shown in Additional file 1.

Putative *qnr *genes were found in 22 out of the 960 genomes tested. The species containing putative *qnr *genes belong to eight different genera (Figure 2). Most of them inhabit an aquatic environment (in some instances the deep sea) suggesting that plasmidic *qnr *may have been originated from aquatic bacteria [17,19,18].

**Figure 3:**
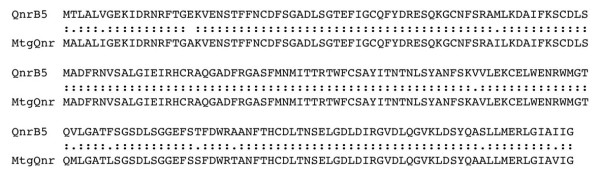
**Alignment of the plasmid-encoded protein QnrB with a putative Qnr protein encoded in a marine metagenome**. The percentage of identity between both proteins was 94.9%, with a homology of 99.5%. Top QnrB5, bottom Qnr protein from a marine metagenome (MtgQnr). Two dots indicate identical amino acids, one dot homologous amino acids.

Since the large majority of bacterial species have not been cultured so far, metagenomic studies allow a more powerful search of relevant genes in different environments by means of non-culture based methodologies. Thus, besides analysing sequenced genomes, available metagenomic sequence databases  were also searched for the presence of *qnr *genes. Our study showed that the metagenomes from sea-water [47] contain genes homologous to *qnr *(hereafter named as Mtg*qnr*), further supporting the notion that *qnr *genes are originated from aquatic microorganisms. One of the *qnr *sequences found in this metagenomic search was highly homologous to plasmid-encoded QnrB [15] proteins (Figure 3). It has been shown that *S. algae *is likely the origin of plasmid-encoded *qnrA *genes [17]. Our analyses, strongly suggest that plasmid-encoded *qnrB *has also originated from a currently unknown, marine microorganism.

The regions surrounding the chromosomal *qnr *genes found in our bioinformatics analysis were analysed to ascertain whether, based in the level of sinteny, the origin of these *qnr *determinants is likely monophyletic or polyphyletic. As shown in Figure 2, and in Additional file 1, the regions are very similar for some of the analysed species belonging to the same genus, although clear differences are also observed for other species.

**Figure 4:**
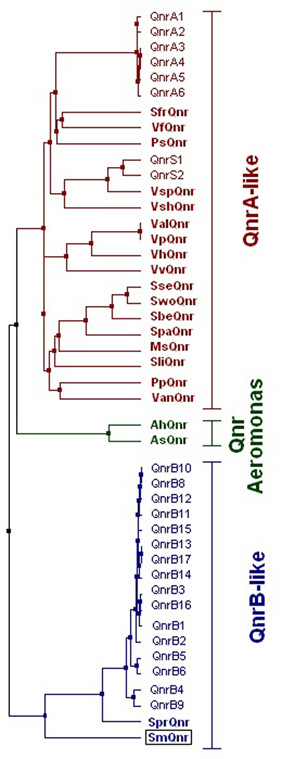
**Clustal analysis of Qnr proteins**. Characterized and predicted Qnr proteins were clustered. The tree was calculated using the average percentage of identity. Other two members of the pentapeptide repeat family with proven resistance to fluoroquinolones, MfpA from *Mycobacterium tuberculosis *and McbG from *Escherichia coli*, were included in the analysis, but are not shown because they plotted out of the Qnr tree. Chromosomally-encoded Qnr proteins are highlighted in bold. The position of the SmQnr protein from *S. maltophilia *as a member of the QnrB cluster is highlighted with a square. Novel Qnr proteins are named according to the species where they are originated: AhQnr (*Aeromonas hydrophila*), AsQnr (*Aeromonas salmonicida*), MsQnr (*Moritella *sp), SprQnr (*Serratia proteamaculans*), SbeQnr (*Shewanella benthica*), SfrQnr (*Shewanella frigidimarina*), SliQnr (*Shewanella loihica*), SpaQnr (*Shewanella pealeana*), SseQnr (*Shewanella sediminis*), SwoQnr (*Shewanella woodyi*), SmQnr (*Stenotrophomonas maltophilia*), PsQnr (*Psychromonas*), ValQnr (*Vibrio alginolyticus*), VanQnr (*Vibrio angustum*), VfQnr (*Vibrio fischeri*), VhQnr (*Vibrio harveyi*), VshQnr (*Vibrio shilonii*), VspQnr (*Vibrio splendidus*), VvQnr (*Vibrio vulnificus*).

For instance, the structure surrounding *qnr *in *Vibrio *is very similar for three species, whereas the structure in five other species and in *Photobacterium profundum *is different. The high level of observed sinteny in these regions, suggests that *qnr *was acquired by *Vibrio parahaemolyticus*, *Vibrio alginolyticus *and *Vibrio harveyi *before their divergence, and was acquired by the other *Vibrionaceae *after their divergence. This idea fits well with the relationship of *Vibrio *Qnr proteins (Figure 4).

Sequence data were inspected for the presence of elements potentially involved in the transfer of *qnr *genes. As shown in Figure 2, putative transposases or integrases were found in the regions surrounding the *qnr *determinants in some *Shewanella *species, and one putative recombinase was found as well in *Moritella *sp. Whether those elements are remnant of former gene transfer events or constitute a risk for the transference of those *qnr *genes through HGT to a new host, remains to be established.

In any case, the analyses of available sequences of bacterial genomes suggest that *qnr *genes are ancient elements in the bacterial chromosomes of a specific subset of bacterial species. Noteworthy, those genes are frequently flanked by genes coding for putative efflux pumps (Figure 2), a situation that suggests that Qnr may have functional roles in detoxification processes in water-dwelling bacteria. Although this linkage has not been so far observed for plasmid-encoded *qnr *genes, the cooperation of two different mechanisms of resistance might be an important element in current resistance to quinolones.

### A putative qnr gene is present in the chromosome of S. maltophilia

Once the bioinformatic analysis was made, we focused our efforts in the study of the organism with highest clinical relevance. Among the organisms carrying putative uncharacterized *qnr *genes we chose *S. maltophilia*, which is an intrinsically resistant opportunistic pathogen responsible for 4.3% of Gram-negative infections among intensive care units patients in the USA [48].

Noteworthy, in *S. maltophilia *resistance to fluoroquinolones (particularly to less active ones, such as norfloxacin) is not always accompanied by high level resistance to nalidixic acid, which is the usual and classical association in Enterobacteria. Interestingly, resistance to fluoroquinolones and susceptibility to nalidixic acid in *S. maltophilia *resemble the phenotype recently observed by Cano ME *et al *(unpublished results) in some *Enterobacteriaceae *containing *qnr *genes. A putative *qnr *gene (Sm*qnr*) was identified in the two available full-genome sequences of *S. maltophilia*: the clinical isolate K279a [49], and the environmental strain R551-3, . As shown in Figure 2, the genomic structure around Sm*qnr *was the same in both strains, in spite their different (clinical and environmental) source of isolation.

**Figure 5:**
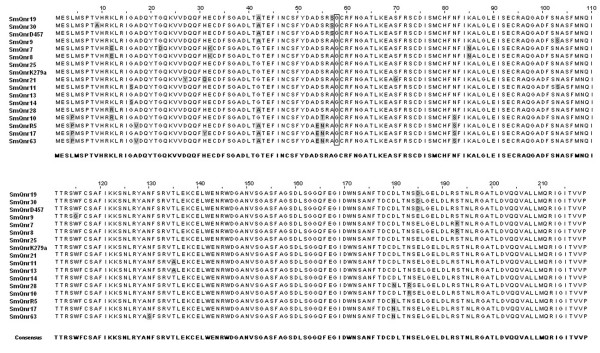
**Alignment of deduced amino acid sequences of different SmQnr proteins**. The amino acids that changed in the different SmQnr alleles are highlighted in a grey square. The glycine residue (G) separating the two domains of pentapetide repeats, is marked with a box.

This high degree of sinteny, together with the lack of elements involved in HGT near Sm*qnr *suggests that this gene has an ancient origin in *S. maltophilia*. Further confirmation of this likely monophyletic origin has been obtained from the analyses of the Sm*qnr *sequence from different *S. maltophilia *isolates in this study. The predicted translation of Sm*qnr *sequence is a 219 amino acid protein (Figure 5), containing tandem repetitions of the pattern (A/C/S/T/V)(D/N)(L/F)(S/T/R)(G/R) typical of the pentapeptide family [20,18,22], with two domains of pentapeptide repeats separated by a single glycine, that corresponds to a COG1357 motif.

### Prevalence of the Smqnr gene in Stenotrophomonas maltophilia strains

Since Sm*qnr *might be involved in quinolone resistance, we wanted to ascertain the prevalence of this gene in *S. maltophilia *populations. To that goal, and since the sequences of the *qnr *genes of the strains K279a and R551-3 were slightly different, two pairs of oligonucleotides QnrM+/- and QnrMR55+/- were designed to amplify Sm*qnr *genes from different *S. maltophilia *strains. The set of analysed strains comprised both clinical and environmental *S. maltophilia *isolates (Table 1).

**Table 1:** Strains and plasmids used in this work.

Strain or plasmid	Description	**Reference or source**
*Escherichia coli*		
KZM120	Δ*acrAB*::Tn903 Kan^r^	[66]
*S. maltophilia*		
C357	Urinary isolate	[67]
CO47	Clinical	Lab collection
CO48	Bronchial aspirate isolate	[67]
D388	Urinary isolate	[67]
D457	Bronchial aspirate isolate	[38]
E301	Urinary isolate	[67]
E539	Infected wound isolate	[67]
E729	Urinary isolate	[67]
E759	Sputum isolate	[67]
E824	Blood	[67]
E847	Clinical	[39]
E923	Sputum isolate	[67]
E999	Respiratory secretion isolate	[67]
F227	Blood culture isolate	[67]
F375	Blood culture isolate	[67]
F861	Sputum isolate	[67]
G51	Blood culture isolate	[67]
e-a21	Sewage (Braunschweig, Germany)	[68]
e-a63	Sewage (Braunschweig, Germany)	Provided by Gabrielle Berg
e-p5	Rhizosphere of Brassica napus L. (Rostock, Germany)	[68]
R551-3	Environmental	Provided by Daniel van der Lelie

**Plasmids**		

pGEM-T	Cloning vector with polyA, amp^r^	Promega
pBS3.7	pGEM-T with *Smqnr *of *S. maltophilia *E759	This work
pBS3.8	pGEM-T with *Smqnr *of *S. maltophilia *E923	This work
pBS3.9	pGEM-T with *Smqnr *of *S. maltophilia *E999	This work
pBS3.10	pGEM-T with *Smqnr *of *S. maltophilia *G51	This work
pBS3.11	pGEM-T with *Smqnr *of *S. maltophilia *E539	This work
pBS3.13	pGEM-T with *Smqnr *of *S. maltophilia *D388	This work
pBS3.14	pGEM-T with *Smqnr *of *S. maltophilia *CO48	This work
pBS3.17	pGEM-T with *Smqnr *of *S. maltophilia *C357	This work
pBS3.19	pGEM-T with *Smqnr *of *S. maltophilia *F375	This work
pBS3.25	pGEM-T with *Smqnr *of *S. maltophilia *E847	This work
pBS3.28	pGEM-T with *Smqnr *of *S. maltophilia *CO47	This work
pBS3.30	pGEM-T with *Smqnr *of *S. maltophilia *E824	This work
pBS3.D457	pGEM-T with *Smqnr *of *S. maltophilia *D457	This work
pBS3.R5	pGEM-T with *Smqnr *of *S. maltophilia *R551-3	This work
pBS3.21	pGEM-T with *Smqnr *of *S. maltophilia *e-a21	This work
pBS3.63	pGEM-T with *Smqnr *of *S. maltophilia *e-a63	This work

The SmQnr sequences from the different strains display a high amino acid identity, ranking from 94 to 100% (Figure 5). However the amino acid identity of the SmQnr protein encoded in the chromosome of the sequenced *S. maltophilia *strain K279a, is low compared with other known Qnr proteins, sharing 38%, 59%, 38% and 41% with QnrA1 (AY070235), QnrB2 (DQ351241), QnrS2 (AB187515) and VvQnr (DQ889870) respectively. For this reason we consider that Sm*qnr *represents a possible new subtype of *qnr *genes. Clustal analysis (Figure 4) indicates that the SmQnr protein clusters with plasmidic QnrB proteins, although forms a separate branch. Therefore, our results suggest that SmQnr is the first characterized chromosomally encoded QnrB-like protein.

We were unable to amplify the full *qnr *gene from the isolates E729, F227, E301, F861 and e-p5. Since *qnr *sequences where slightly different in K279a and R551-3, as well as in the isolates analysed in the present study, two new pairs of oligonucleotides (qnrI1/qnrI2 and qnrI3/qnrI4) were used to amplify an internal conserved region of Sm*qnr*. Positive amplification was obtained in all cases (not shown) indicating that all *S. maltophilia *isolates contained the *qnr *gene.

### Functional analysis of SmQnr as a quinolone resistance determinant in a heterologous host

To ascertain whether SmQnr might contribute to quinolone resistance in a heterologous host, the different alleles of the *qnr *gene obtained from different *S. maltophilia *strains were cloned into the plasmid pGEM-T. Corresponding recombinant plasmids, containing the Sm*qnr *gene, were expressed in *E. coli*, and the quinolone susceptibility of strains expressing SmQnr was compared to that of the isogenic *E. coli *strain. We found that *in trans *expression of SmQnr from different recombinant plasmid results in a 2 to 32-fols decrease in the quinolone susceptibility of *E. coli *(Table 2).

**Table 2:** MICs (μg/ml) of quinolones for *E. coli *KZM120 carrying different *Smqnr *genes from *S. maltophilia *isolates.

**Plasmids**	**Antibiotics**									
	
	**NAL**	**NOR**	**SPA**	**CIP**	**ENX**	**LEV**	**GAR**	**GRP**	**TRV**	**MOX**
pGEM-T	1	0.016	0.032	0.002	0.032	0.004	0.002	0.001	0.004	0.004
pBS3.7	1	0.016	0.032	0.004	0.064	0.008	0.016	0.004	0.016	0.008
pBS3.8	1	0.032	0.032	0.008	0.064	0.016	0.032	0.008	0.032	0.016
pBS3.9	1	0.016	0.032	0.004	0.064	0.008	0.016	0.016	0.032	0.016
pBS3.10	1	0.016	0.032	0.004	0.064	0.008	0.008	0.004	0.016	0.016
pBS3.11	1	0.016	0.032	0.004	0.064	0.008	0.004	0.004	0.008	0.008
pBS3.13	1	0.016	0.032	0.004	0.064	0.008	0.008	0.004	0.016	0.016
pBS3.14	1	0.016	0.032	0.004	0.064	0.008	0.008	0.004	0.016	0.016
pBS3.17	1	0.016	0.032	0.004	0.064	0.008	0.016	0.008	0.016	0.016
pBS3.19	1	0.016	0.032	0.004	0.064	0.008	0.008	0.004	0.016	0.008
pBS3.25	1	0.016	0.032	0.004	0.064	0.008	0.064	0.016	0.032	0.032
pBS3.28	1	0.016	0.032	0.004	0.064	0.008	0.004	0.002	0.008	0.008
pBS3.30	1	0.016	0.032	0.004	0.064	0.008	0.004	0.002	0.008	0.008
pBS3.R5	1	0.016	0.032	0.004	0.064	0.008	0.008	0.002	0.016	0.008
pBS3.21	1	0.016	0.032	0.004	0.064	0.008	0.016	0.004	0.016	0.016
pBS3.63	1	0.016	0.032	0.004	0.032	0.008	0.008	0.002	0.008	0.008

NAL; nalidixic acid, NOR; norfloxacin, SPA; sparfloxacin, CIP; ciprofloxacin, ENX; enoxacin, LEV; levofloxacin, GAR; garenoxacin, GRP; grepafloxacin, TRV; trovafloxacin, MOX; moxifloxacin.

**Figure 6:**
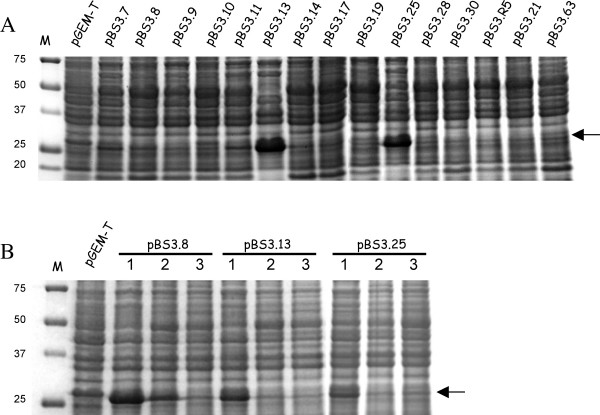
**Estimation of the expression of different SmQnr alleles in *E. coli***. M, molecular mass standards, lane 2, *E. coli *KZM120 (Δ*acrAB*) carrying pGEM-T (negative control), (A) Expression of SmQnr by *E. coli *strains containing different plasmids that encode different SmQnr alleles. The SmQnr protein used for its identification by MS-MALDI-TOF (see Methods) was the allele encoded by pBS3.13. (B) To establish the effect of the level of expression of SmQnr on the susceptibility to antibiotics, the original clone (1) and two re-transformants (2 and 3) were analysed for each of the plasmids pBS3.8, pBS3.13 and pBS3.25. The position of SmQnr is indicated with an arrow.

Noteworthy, different recombinant strains presented slight, but consistent, differences in their susceptibility to several quinolones (Table 2). It has been previously stated that transconjugants of plasmids containing the *qnr *gene present disparate levels of quinolone resistance, likely due to different levels of expression of the Qnr protein in the different transconjugants [50]. To ascertain whether this could be the situation with the different SmQnr-expressing plasmids, the expression of the SmQnr protein was estimated in the different *E. coli *transformants. As shown in Figure 6A, transformants containing either pBS3.25 or pBS3.13 expressed higher levels of SmQnr than the other transformants.

During our work, we detected that some Qnr-expressing plasmids were lost after subculturing even in the presence of ampicillin, although they could be maintained in the presence of carbenicillin. Plasmid loss after the bacteria has degraded the selective antibiotic is an indication of physiological burden [51]. Our results suggest that high-level expression of SmQnr is likely harmful for *E. coli*. It is possible that, as described for other systems [52], a fast adaptation between the host cell and the plasmid occurred that results in reduced levels of SmQnr and that this allowed some of the clones to overcome the putative SmQnr toxicity. To address this possibility, *E. coli *KZM120 was retransformed with the plasmids pBS3.8, pBS3.25 and pBS3.13. Two different clones from each retransformation were chosen and their susceptibility to quinolones tested. As shown in Table 3, an overall reduction in MICs as well as in the level of SmQnr expression (Figure 6B) was observed for the new transformants containing the plasmids pBS3.8, pBS3.25 and pBS3.13. However, the phenotype of reduced susceptibility to quinolones was maintained. Increased resistance due to higher expression of antibiotic resistance genes has been described mainly associated with plasmidic beta-lactamases [53-55]. Our results together with already published data indicate that gene-dosage might be relevant for *qnr*-mediated quinolone resistance.

**Table 3:** MICs (μg/ml) of quinolones for different colonies of *E coli *KZM120 containing each of the plasmids pBS3.8, pBS3.25 and pBS3.13

**Plasmids**	**Antibiotics**									
	
	**NAL**	**NOR**	**SPA**	**CIP**	**ENX**	**LEV**	**GAR**	**GRP**	**TRV**	**MOX**
pGEM-T	1	0.016	0.032	0.002	0.032	0.004	0.002	0.001	0.004	0.004
pBS3.8 1	1	0.032	0.032	0.008	0.064	0.016	0.032	0.008	0.032	0.016
pBS3.8 2	1	0.016	0.032	0.004	0.064	0.008	0.016	0.008	0.032	0.016
pBS3.8 3	1	0.016	0.032	0.004	0.064	0.008	0.016	0.008	0.032	0.016
pBS3.13 1	1	0.016	0.032	0.004	0.064	0.008	0.008	0.004	0.016	0.016
pBS3.13 2	1	0.016	0.032	0.004	0.064	0.008	0.002	0.002	0.008	0.008
pBS3.13 3	1	0.016	0.032	0.004	0.064	0.008	0.002	0.002	0.008	0.008
pBS3.25 1	1	0.016	0.032	0.004	0.064	0.008	0.064	0.016	0.032	0.032
pBS3.25 2	1	0.016	0.032	0.004	0.064	0.008	0.008	0.004	0.016	0.008
pBS3.25 3	1	0.016	0.032	0.004	0.064	0.008	0.008	0.004	0.016	0.008

Three colonies were tested for each plasmid.

NAL; nalidixic acid, NOR; norfloxacin, SPA; sparfloxacin, CIP; ciprofloxacin, ENX; enoxacin, LEV; levofloxacin, GAR; garenoxacin, GRP; grepafloxacin, TRV; trovafloxacin, MOX; moxifloxacin.

## Conclusion

By using a combination of bioinformatics and functional tools, we found a number of novel putative *qnr *genes in the chromosomes of aquatic bacteria and in metagenomes from marine organisms. This further supports the notion that the origins of *qnr *determinants are in water-dwelling bacteria. It has been recently shown that *Aeromonas *spp. obtained from water samples contain plasmid-encoded *qnr *genes [16]. These results have been interpreted as a consequence of the presence of quinolones in rivers that drive the evolution of aquatic bacteria towards quinolone resistance. This explanation is likely true for plasmid-encoded *qnr *genes. However, the presence of *qnr*-like determinants with conserved genetic environments in the chromosomes of water-borne bacteria, together with the fact that at least for *S. maltophilia *the *qnr *gene is present in all strains, suggests that their presence in bacterial chromosomes is not the consequence of recent HGT events due to the selective pressure of quinolones. Thus, we would expect an ecological role in aquatic environments for those *qnr*-like determinants in addition to quinolone resistance.

The study of the *S. maltophilia *Sm*qnr *gene indicates that SmQnr confers quinolone resistance when expressed from a plasmid in a heterologous host, highlighting the risk of those elements for future development of novel plasmid-mediated Qnr resistance. Altogether, our data supports the reliability of using a predictive approach for analysing antibiotic resistance elements that may potentially disseminate among bacterial populations.

## Authors' contributions

All the authors contributed to write the article. JLM and LMM designed the work. AH and MBS contributed equally to the experimental and bioinformatic work. All authors read and approved the final version of the manuscript.

## Supplementary Material

Additional file 1 Microsoft excel document containing the descriptions of the genes shown in Figure 2. Click here for file
